# Deafness in occludin-deficient mice with dislocation of tricellulin and progressive apoptosis of the hair cells

**DOI:** 10.1242/bio.20147799

**Published:** 2014-07-25

**Authors:** Shin-ichiro Kitajiri, Tatsuya Katsuno, Hiroyuki Sasaki, Juichi Ito, Mikio Furuse, Shoichiro Tsukita

**Affiliations:** 1 Department of Otolaryngology, Head and Neck Surgery, Kyoto University Graduate School of Medicine, Kyoto University, Sakyo-ku, Kyoto 606-8507, Japan; 2 Department of Cell Biology, Faculty of Medicine, Kyoto University, Sakyo-ku, Kyoto 606-8501, Japan; 3 Department of Physical Therapy, Faculty of Community Health Care, Teikyo Heisei University, Uruido Minami, Ichihara, Chiba 290-0193, Japan; 4 Department of Cell Biology, Division of Cell Biology, Kobe University Graduate School of Medicine, Kusunoki-cho, Chuo-ku, Kobe 650-0017, Japan; 5 Division of Cerebral Structure, National Institute for Physiological Sciences, Okazaki, 444-8787, Japan

**Keywords:** Occludin, Deafness, Tight junction, Cochlea, Hair cell, Tricellulin

## Abstract

Occludin is the first identified protein in the tight junction (TJ), but its function has remained for the most part obscure. TJs have been demonstrated to play important roles in the inner ear function, and occludin is expressed in all the epithelial TJs in the inner ear. Thus, we examined the inner ears of occludin-deficient (*Occ^−/−^*) mice. Although inner ears initially developed normally in *Occ^−/−^* mice, apoptosis occurs in hair cells in the organ of Corti around day 12 after birth, and deafness develops. Since hair cell degeneration was not observed in cochlear explant cultures of *Occ^−/−^* mice, environmental changes were considered to be the trigger of cell death. As for the vestibular system, both the morphologies and functions are normal in *Occ^−/−^* mice. These phenotypes of *Occ^−/−^* mice are very similar with those of claudin-14 or claudin-9 deficient mice, leading us to speculate on the existence of imbalance induced by TJ abnormalities, such as localized ionic components. Moreover, the occludin deficiency led to dislocalization of tricellulin, a gene responsible for human deafness DFNB49. The deafness in *Occ^−/−^* mice may be due to this dislocalization of tricellulin.

## INTRODUCTION

Multicellular organisms are composed of various compartments, and maintaining the environments of each compartment is essential for organs to fulfill their functions. In the inner ear, the different environments of endolymph and perilymph must be maintained (reviewed by Wangemann and Schacht, 1996). The endolymph and the perilymph are delineated by epithelial cells, and the leakage of solutes through a paracellular pathway is prevented by tight junctions (TJs) (Kitajiri et al., 2004b; Kitajiri et al., 2004c).

This TJ barrier has been demonstrated to play important roles in the inner ear. First, it has been revealed that claudin-14, a member of the claudin family, which is involved in the barrier function of TJs, is expressed in the organ of Corti of the cochlea (Kitajiri et al., 2004c) and is a gene responsible for human hereditary deafness DFNB29 (Wilcox et al., 2001). Knockout mice of claudin-14 were demonstrated to develop deafness (Ben-Yosef et al., 2003). In addition, claudin-11 knockout mice developed deafness due to selective disruption of the barrier function with disappearance of TJs between basal cells of the stria vascularis (Gow et al., 2004; Kitajiri et al., 2004b). Tricellulin, a TJ transmembrane protein, was also identified as a responsible gene for human hereditary deafness DFNB49 (Chishti et al., 2008; Riazuddin et al., 2006) and knockin mice of tricellulin mutant gene, which mimics one of DFNB49-associated mutations, exhibited profound deafness (Nayak et al., 2013). Furthermore, a mutation in claudin-9 gene, another claudin member expressed in the organ of Corti (Kitajiri et al., 2004c), causes a severe deafness in mice (Nakano et al., 2009).

Occludin is a TJ membrane protein discovered earlier than claudin (Furuse et al., 1993), and is expressed in all the epithelial TJs, including the cochlea and the vestibule in the inner ear (Kitajiri et al., 2004c). However, its function remains unknown since no findings have been reported concerning its involvement in TJs barrier function (Saitou et al., 1998). Occludin knockout mice have been found to develop morphological abnormalities in various organs (Saitou et al., 2000). TJs may contribute to the morphogenesis of tissues through intercellular adhesion since intercellular interactions and adhesions, which organize the tissues, are essential for maintaining functions and morphogenesis of the tissues. In human, mutations of occludin cause brain calcification and renal dysfunction (O'Driscoll et al., 2010; LeBlanc et al., 2013).

The inner ear is considered useful for examining TJs and morphogenesis. In the organ of Corti of the cochlea, hair cells surrounded by supporting cells generate four clear rows (1 row by inner hair cells, 3 rows by outer hair cells), and stereocilia of each hair cell are arranged in an orderly fashion. This morphology provides a model to examine planner cell polarity in mammals (Montcouquiol et al., 2003), and the inner ear is a good target organ to observe morphogenesis. Differentiation and morphogenesis of the inner ear can be examined using expression markers (Hasson et al., 1995), and the period, during which functional development is completed after birth and environmental differences between endolymph and perilymph are generated, has been studied in detail (Lim and Anniko, 1985; Yamasaki et al., 2000). This information is valuable for examining TJ function, which is closely associated with the environments of endolymph and perilymph. Moreover, objective assessments of inner ear functions, including auditory perception and sense of equilibrium, can also be made on mice (Zheng et al., 1999; Iwashita et al., 2001).

Thus, in this study, inner ears of occludin knockout mice were examined in detail to further understand the TJ function in the inner ear.

## RESULTS

### Occludin deficiency causes deafness

**Fig. 1.**
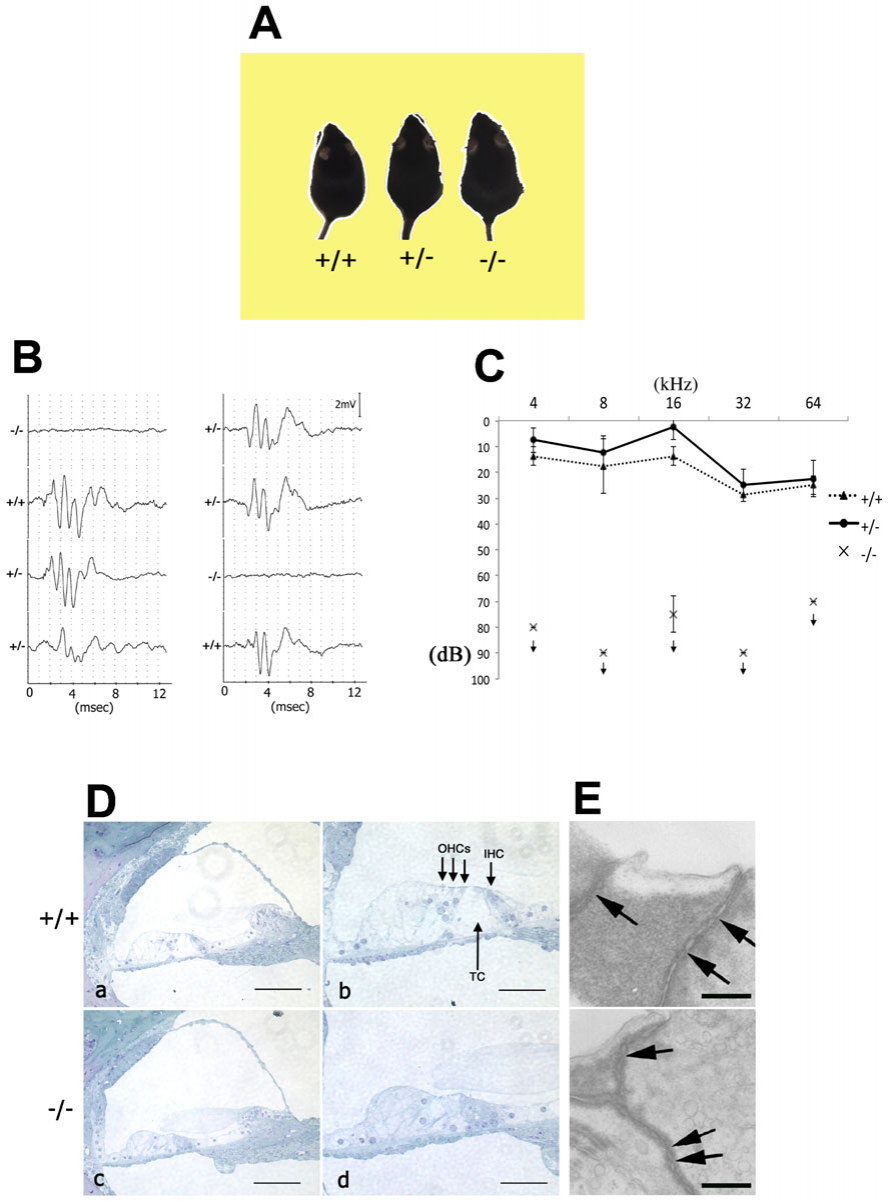
Deafness and structures of the organ of Corti in 6-week-old *Occ^−/−^* mice. (A–C) Deafness in *Occ^−/−^* mice. (A) Loss of Preyer's reflex in *Occ^−/−^* mice. Time-lapse photography captures Preyer's reflex in *Occ^+/+^* and *Occ^+/−^* mice, but not in *Occ^−/−^* mice. Two successive frames after a loud handclap (1-sec interval) were superimposed. These are 6-week-old mice (*Occ^+/−^* intercross littermates). (B) Auditory brainstem response (ABR) to stimuli of 70-decibel (dB) sound pressure level (20 kHz) in two sets of *Occ^+/−^* intercross littermates (6 weeks old). The *Occ^+/+^* and *Occ^+/−^* mice show typical ABR waveform, but the waveform could not be seen in *Occ^−/−^* mice. (C) Hearing thresholds of 6-week-old *Occ^+/+^*, *Occ^+/−^* and *Occ^−/−^* mice at various sound frequencies. These data clearly indicate that the *Occ^−/−^* mice suffered from profound deafness. (D,E) Structures of the organ of Corti in 6-week-old *Occ^−/−^* mice. (D) Toluidine-blue stained Epon semi-thin sections of the cochlea. No gross morphological changes were observed between *Occ^+/+^* and *Occ^−/−^* cochlear duct (a,c), but in higher magnification, outer hair cells (OHCs), inner hair cell (IHC) and the tunnel of Corti (TC) seems to be lost in the organ of Corti in *Occ^−/−^* mice (b,d). (E) Transmission electron micrographs of cell–cell border in the apical surface of the organ of Corti. Kissing points, where tight junction strands between adjacent cells causing occlusion of plasma membrane, can be observed both in the *Occ^+/+^* and *Occ^−/−^* mice (arrows). Scale bars: 100 µm (Da,Dc), 50 µm (Db,Dd), 0.3 µm (E).

We previously generated occludin-deficient (*Occ^−/−^*) mice. In these *Occ^−/−^* mice, the barrier function of intestinal epithelium was normal, but histological abnormalities were found in several tissues (Saitou et al., 2000). Interestingly, they showed no Preyer's reflex, a motor reflex in response to auditory stimuli. When a sound stimulus was administrated in the form of a loud handclap, they showed no reflexive reaction (Fig. 1A). We then measured the auditory brainstem response (ABR) to stimuli of 70-decibel (dB) (20 kHz) sound pressure level (SPL) in two sets of *Occ^+/−^* intercross littermates (6 weeks old), which were later genotyped (Fig. 1B). Among 8 littermates in total, two showed no ABR, while the others showed a typical ABR waveform. Interestingly, only the two littermates showing no ABR were identified as *Occ^−/−^* mice. This perfect correlation between *Occ^−/−^* genotype and the lack of ABR was reproducibly obtained in different series of measurements. In Fig. 1C, the hearing thresholds of 6-week-old mice were measured at various sound frequencies. *Occ^+/+^* and *Occ^+/−^* mice showed normal hearing thresholds (10–50 dB SPL), while *Occ^−/−^* mice showed profound deafness (hearing threshold, >70–90 dB SPL).

### Occludin deficiency causes degeneration of the organ of Corti

**Fig. 2.**
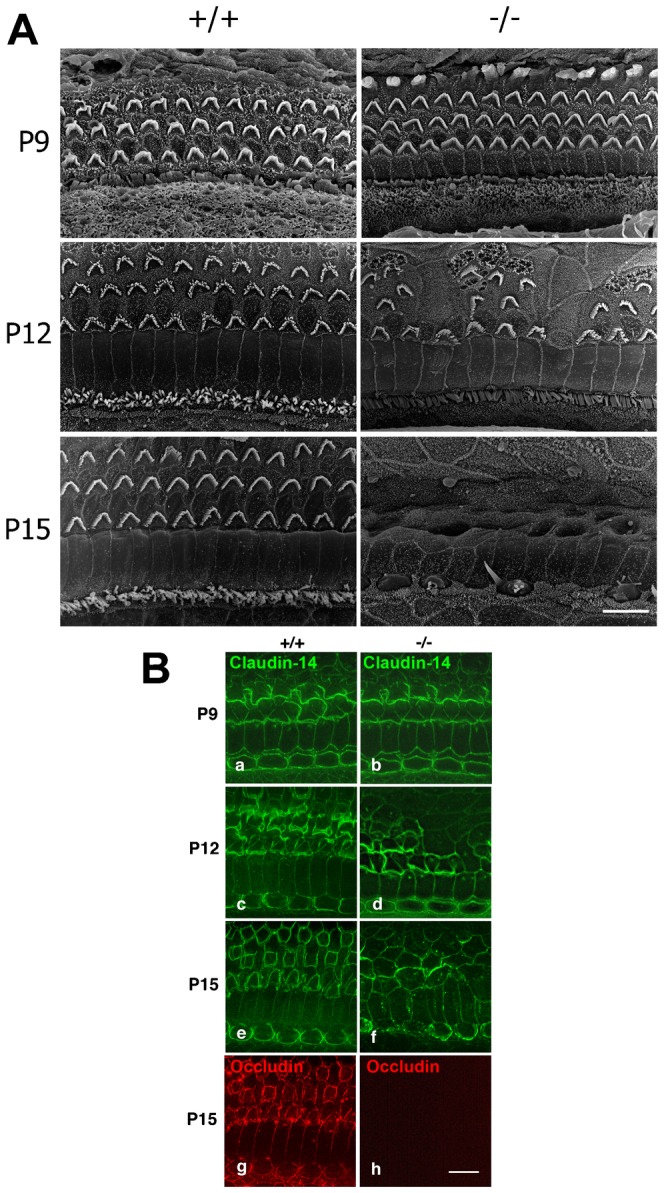
Scanning electron micrographs of the organ of Corti and expression of claudin-14 in the organ of Corti. (A) Scanning electron micrographs of the organ of Corti. Up to postnatal day 9 (P9), both inner and outer hair cells appeared to develop normally in *Occ^−/−^* mice. However, at postnatal day 12 (P12), outer hair cells began to disappear from the surface of the *Occ^−/−^* organ of Corti, and be replaced by supporting cells. At postnatal day 15 (P15), the outer hair cells had disappeared almost entirely, and also the inner hair cells showed changes, and began to disappear from the surface. (B) Expression of claudin-14 in the organ of Corti. The whole mount double immunofluorescence micrographs for claudin-14 (a–f, green) and occludin (g,h, red) of the *Occ^+/+^* and *Occ^−/−^* organ of Corti. In every stage of degeneration (P9–P15; postnatal days 9–15), claudin-14 was expressed normally at the junctional complex along the apical borders of the organ of Corti even in *Occ^−/−^* mice. Scale bars: 10 µm.

Light microscopic observation with toluidine blue-stained Epon sections identified deformity of the organ of Corti in the 6-week-old *Occ^−/−^* mice (Fig. 1D). A collapse of the tunnel of Corti was observed, and outer hair cells were damaged or lost. There was no morphological change in Reissner's membrane, tectorial membrane, spiral ligament or stria vascularis. Then, the apical surface of the Corti organ was observed by scanning electron microscopy (Fig. 2A). Up to 9 days after birth, the *Occ^−/−^* cochlea was indistinguishable from that of the control. However, subsequently rapid loss of OHC was observed.

At 12 days after birth, outer hair cells began to disappear rapidly, and at day 15, the outer hair cells had disappeared almost entirely, and also the inner hair cells showed changes, and began to disappear. These changes and the disappearance of hair cells were considered to be the cause of deafness in *Occ^−/−^* mice.

### No structural changes in TJs of the inner ear were observed in *Occ^−/−^* mice

Whether structural changes in TJs occurred in *Occ^−/−^* mice was examined using transmission electron microscopy, since occludin is a membrane protein localized at TJs (Furuse et al., 1993). However, kissing points, where tight junction strands between adjacent cells causing occlusion of plasma membrane, appeared normal, as in other organs of *Occ^−/−^* mice (Saitou et al., 2000) (Fig. 1E), and TJs were apparently normal also in *Occ^−/−^* mice.

Expression of claudin-14, which is expressed in TJs of the organ of Corti, and its mutations cause deafness (Wilcox et al., 2001; Ben-Yosef et al., 2003), was examined using whole mount immunostaining, but no changes were observed in *Occ^−/−^* mice (Fig. 2B). In addition to claudin-14, claudin-1, -2, -3, -9, -10, -12 and -18 were expressed in the organ of Corti (Kitajiri et al., 2004c), but no change of their expression could be confirmed in *Occ^−/−^* mice (claudin-9 and -12 in supplementary material Fig. S1).

**Fig. 3.**
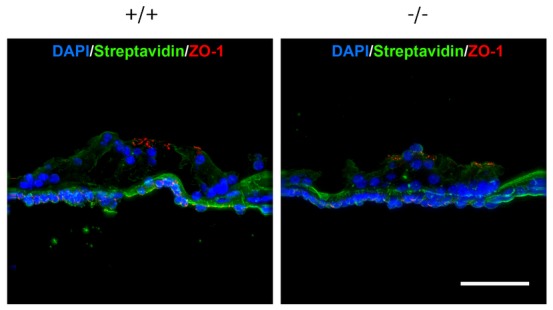
Tracer permeability assay of the organ of Corti of 6-week-old mice. An isotonic solution containing freshly made biotinylation reagent was injected into the perilymph space from the round window of the cochlea, and after 5 minutes incubation followed by being washed with PBS, the cochlea was dissected out, fixed and frozen. Frozen sections were triple stained with anti-ZO-1 Ab in red, streptavidin in green and DAPI in blue to detect tight junctions, bound biotin, and nuclei, respectively. In this study, the basilar membrane barrier that faces the perilymph, not an apical surface of the organ of Corti on which hair cells reside, was examined, and there was no difference between the *Occ^+/+^* and *Occ^−/−^* organ of Corti. n = 3. Scale bar: 50 µm.

To examine the barrier function of the *Occ^−/−^* inner ear, we performed a tracer experiment as described previously (Kitajiri et al., 2004b). The perilymph compartment was perfused from the round to oval windows with an isotonic solution containing a primary amine-reactive biotinylation reagent (*Mr* = 556.59), which is covalently cross-linked to the accessible cell surface. After 5 minutes incubation followed by perfusion with PBS, the cochlea was dissected out, fixed and frozen. Frozen sections of the cochlea were labeled with anti-ZO-1 (TJ scaffold protein) Ab in red and streptavidin in green to detect TJ and bound biotin, respectively (Fig. 3). As a result, there was no difference in the diffusion of tracer between the *Occ^−/−^* organ of Corti and the *Occ^+/+^* organ of Corti. In this study, the basilar membrane barrier that faces the perilymph, not an apical surface of the organ of Corti on which hair cells reside, was examined, and maintenance of the TJs barrier function of the organ of Corti in *Occ^−/−^* mice was indicated. Although occludin was also expressed in marginal cells and basal cells of the stria vascularis, the barrier function of the stria vascularis was not affected either (data not shown). Historically, occludin deficiency does not cause evident loss of barrier function (Saitou et al., 1998), the data in this study have also suggested that occludin deficiency does not affect the TJ structure or barrier function.

### Environmental factors induce apoptosis in hair cells of the *Occ^−/−^* organ of Corti

**Fig. 4.**
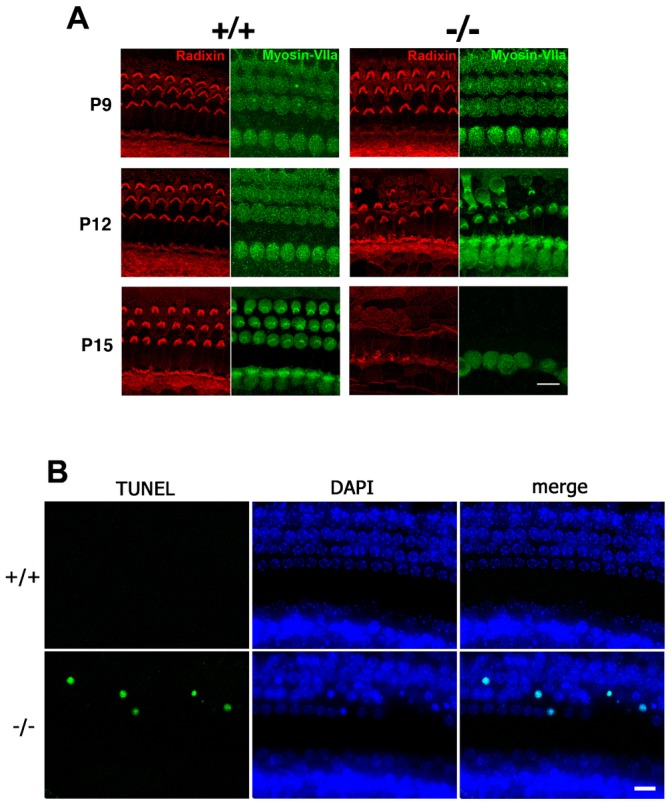
Progressive loss of hair cells in the *Occ^−/−^* organ of Corti. (A) The whole mount organ of Corti were double stained with anti-radixin mAb in red and anti-myosin VIIa pAb in green to detect stereocilia and hair cell bodies, respectively. In good agreement to the scanning electron micrographs (Fig. 2A), the radixin-positive stereocilia of the outer hair cells began to disappear around postnatal day 12 (P12). The radixin-positive stereocilia of the inner hair cells seemed to be also affected around postnatal day 15 (P15). Note that not only stereocilia but myosin VIIa-positive whole hair cell bodies themselves were disappearing. (B) The identification of apoptosis as the mechanism underlying the hair cell death in the *Occ^−/−^* organ of Corti. The whole mount organ of Corti at postnatal day 12, when the outer hair cells in the *Occ^−/−^* mice degenerates, were examined. Nuclei were detected by DAPI in blue. The nuclei of degenerating outer hair cells in the *Occ^−/−^* mice were labeled in green by the TUNEL reaction. n = 3. Scale bars: 10 µm.

For detailed study of morphological changes in the organ of Corti, the organs of Corti undergoing morphogenesis were whole mount stained with markers. Radixin was used as a marker for stereocilia, and myosin VIIa for hair cells (Kitajiri et al., 2004a; Hasson et al., 1995). As a result, the number of hair cells was decreased in the *Occ^−/−^* organ of Corti (Fig. 4A).

To monitor the process of cell death within the organ of Corti, we performed TUNEL experiments in P12, which is on the process of degeneration. As a result, in the *Occ^−/−^* mice, degenerating OHCs were labeled, which was never been seen in the *Occ^+/+^* mice (Fig. 4B).

**Fig. 5.**
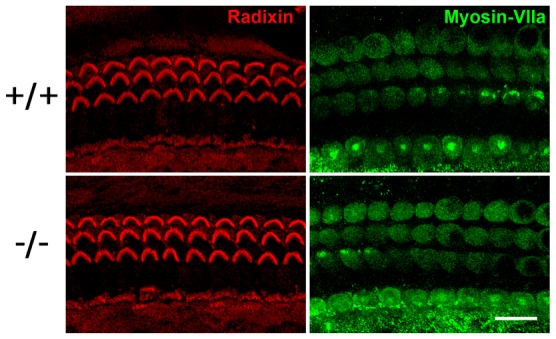
The hair cells of the organ of Corti explants of *Occ^+/+^* and *Occ^−/−^* mice. The organ of Corti explants obtained from P3 mice were maintained in normal culture conditions to P15. The samples were immunostained using the anti-radixin mAb (red) and anti-myosin VIIa pAb (green), to observe stereocilia and hair cell bodies, respectively. In sharp contrast to the organ of Corti of *Occ^−/−^* mice *in vivo* (Fig. 4A), hair cells in the *Occ^−/−^* organ of Corti explants survived up to P15. n = 3. Scale bar: 10 µm.

To clarify whether degeneration of hair cells of *Occ^−/−^* mice is caused by a signal intrinsic to the cell or by extracellular conditions, we maintained explants derived from the organ of Corti of *Occ^−/−^* and *Occ^+/+^* mice for up to 12 days (from P3 to P15) in culture medium. At P15, immunostaining with anti-radixin and anti-myosin VIIa antibodies revealed the survival of explants of *Occ^−/−^* mice hair cells, as observed with the explants of *Occ^+/+^* mice (Fig. 5). This is in sharp contrast to the organ of Corti of *Occ^−/−^* mice *in vivo*, in which most of the outer hair cells and some inner hair cells had disappeared by P15 (Fig. 2A, Fig. 4A). These findings suggest that the apoptosis in hair cells of *Occ^−/−^* mice was induced by some environmental factors.

### *Occ^−/−^* mice have normal vestibular function

**Fig. 6.**
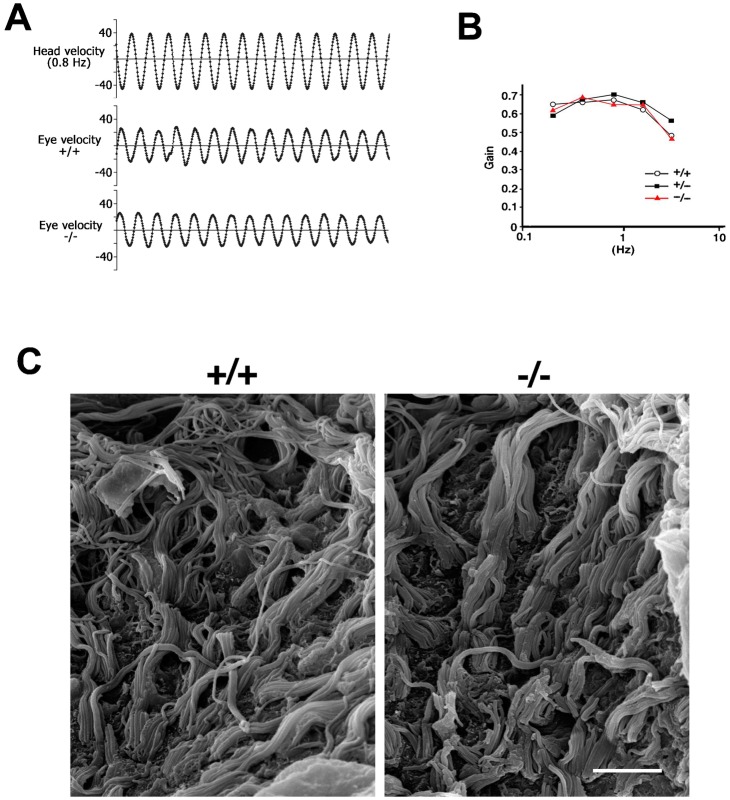
The balance function of 6-week-old *Occ^−/−^* mice. (A) The vestibulo-ocular reflex to 0.8 Hz sinusoidal head rotation. The eye position during the head rotation stimulus was recorded by a CCD camera and the eye velocity was calculated. The sinusoidal curves of eye velocities were indistinguishable between *Occ^+/+^*, *Occ^+/−^* and *Occ^−/−^* mice. (B) The gain was obtained by dividing the peak eye velocity by the peak head velocity. The vestibulo-ocular reflex gains of *Occ^−/−^* mice are also normal at any frequency of the head rotation stimulus in *Occ^+/+^* and *Occ^−/−^* mice. (C) Scanning electron micrographs of the 6-week-old crista ampullaris of the vestibule. The appearance of stereocilia and hair cells are totally normal in *Occ^−/−^* mice. Scale bar: 5 µm.

The expression of occludin is also observed in TJs in the vestibule (Kitajiri et al., 2004c), leading us to speculate on a possible imbalance in *Occ^−/−^* mice. Thus, we measured the vestibulo-ocular reflex. A mouse was mounted on a turntable and rotated sinusoidally, and the eye position was recorded by a CCD camera. However, as shown in Fig. 6A, the sinusoidal curve of eye velocity shows no difference among *Occ^+/+^*, *Occ^+/−^*, or *Occ^−/−^* mice. Indeed, the vestibulo-ocular reflex gain of the *Occ^−/−^* mouse was normal at all frequencies of head rotation stimulus (Fig. 6B). Scanning electron microscopy consistently revealed that the appearance of sensory epithelia in the crista ampularis was indistinguishable between *Occ^+/+^* and *Occ^−/−^* mice (Fig. 6C).

### Occludin deficiency leads to dislocalization of tricellulin in cochlea

Tricellulin is a recently identified constituent of TJ, and is the first marker of the tricellular tight junction (tTJ) where three epithelial cells meet in polarized epithelia (Ikenouchi et al., 2005). It has COOH-terminal sequence similar to occludin, and is necessary to maintain the epithelial barrier (Ikenouchi et al., 2005). The mutations of tricellulin were reported to cause human deafness DFNB49 (Riazuddin et al., 2006), and occludin was reported to support tricellular localization of tricellulin (Ikenouchi et al., 2008). The importance of tricellular localization of tricellulin is also suggested by human deafness DFNB42 (Borck et al., 2011). The responsible gene of DFNB42 encodes ILDR-1, which is required for the localization of tricellulin at tricellular contacts (Higashi et al., 2013).

**Fig. 7.**
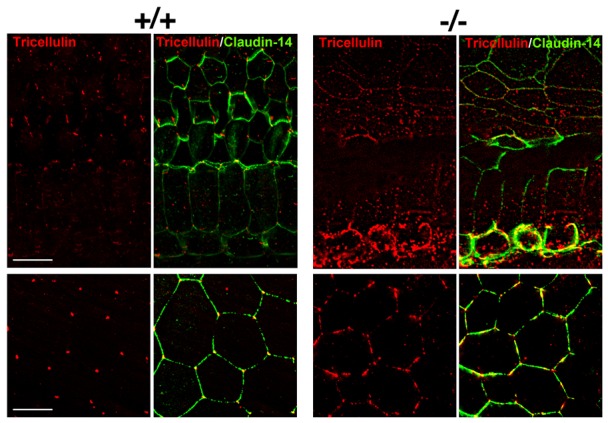
The dislocalization of tricellulin in *Occ^−/−^* cochlea. The organ of Corti (upper panels) and stria vascularis Corti (lower panels) of P15 *Occ^+/+^* and *Occ^−/−^* mice were labeled with anti claudin-14 (green) and anti tricellulin (red) antibodies. In *Occ^+/+^* tissue tricellulin is mainly detected in tTJs, whereas in occludin-deficient tissue tricellulin is found in bTJs in addition to tTJs. Scale bars: 10 µm.

Thus, we examined if occludin deficiency cause dislocalization of tricellulin in cochlea. Two parts of cochlea, stria vascularis and organ of Corti, were dissected out from *Occ^+/+^* and *Occ^−/−^* mice, and whole mount double immunofluorescence staining were performed (Fig. 7). In *Occ^+/+^* cochlea, tricellulin was localized at tTJ, as reported previously (Riazuddin et al., 2006). But in *Occ^−/−^* cochlea, tricellulin was mislocalized and its signal at bicellular TJs was increased. The dislocalization of tricellulin was observed in other tissues of *Occ^−/−^* mice (supplementary material Fig. S2).

## DISCUSSION

Occludin is a molecule that was first identified as a membrane protein of TJs, but its functions remain unclear (Furuse et al., 1993; Saitou et al., 1998; Saitou et al., 2000). TJs were demonstrated to play roles in compartmentalization in multicellular organisms (Schneeberger and Lynch, 1992; Anderson and Van Itallie, 1995; Balda and Matter, 1998; Tsukita et al., 2001), and detailed study of its physiological functions requires functional analysis in living tissues. In the inner ear, there are compartments of endolymph and perilymph, and maintenance of the environmental differences between them is essential for inner ear functions (reviewed by Wangemann and Schacht, 1996). Functional assessments were made in the inner ear, for which TJs play important roles and detailed assessments of functions and morphology can be made (Kitajiri et al., 2004b).

Although inner ears initially developed normally in *Occ^−/−^* mice, apoptosis occurs in outer hair cells in the organ of Corti followed by in inner hair cells, and deafness developed. Cell death occurred at around day 12 after birth in cochlear hair cells, the same period when the ionic environment in the endolymph and high resting endocochlear potential (EP) appear (Yamasaki et al., 2000). Since hair cell degeneration was not observed in cochlear explant cultures of *Occ^−/−^* mice, environmental changes were considered to be the trigger of cell death. As no abnormalities were observed in the vestibula, in which high resting potential comparable with EP dose not exist, there is a possibility that cochlear hair cells of *Occ^−/−^* mice cannot tolerate the high EP.

Occludin is not essential for TJ formation (Saitou et al., 1998), and the mice in this study underwent no structural changes in the TJs. Since morphological abnormalities of many organs were observed in the occludin knockout mice, occludin is considered to be essential for maintaining the morphology of other organs (Saitou et al., 2000). There has been some reports that occludin is involved in the regulation of TJ permeability in response to cytokines (Marchiando et al., 2010; Van Itallie et al., 2010), suggesting that occludin play a role in the modulation of barrier function. Recently, it has been reported that mutations of occludin cause brain calcification and renal dysfunction (O'Driscoll et al., 2010; LeBlanc et al., 2013), which further suggests the occludin involvement in TJ barrier function.

Generation of mice lacking claudin-14, a membrane protein expressed in the organ of Corti, was reported (Ben-Yosef et al., 2003). In these mice, hair cells in the organ of Corti initially developed normally, but degenerated within 3 weeks after birth and developed deafness. However, the EP was not reduced, and collapse of the barrier function has not been demonstrated. Mice harboring a mutation in claudin-9 gene, which is another component of TJ in the organ of Corti, also exhibit severe deafness with progressive degeneration of hair cells (Nakano et al., 2009). The phenotypes of these mice are very similar with those of *Occ^−/−^* mice in the following points: morphological changes of the organs of Corti, preceding degeneration in outer hair cells, degeneration timing, and no degeneration observed in explant cultures. In addition, since the expression of claudin-14 and claudin-9 is maintained in the cochleae in *Occ^−/−^* mice, occludin deficiency does not cause changes in the expression or localization of claudin-14 or claudin-9, resulting in the same abnormalities. Considering that three mutant mice lacking TJ components exhibit similar phenotype, some defect in TJ function would be the cause of hair cell degeneration, leading to hearing loss.

It has been reported that tricellulin fail to localize at tricellular tight junctions (tTJs) in the occludin-knockdown cells (Ikenouchi et al., 2008), although no change was observed in the report from another group (Van Itallie et al., 2010). Our *in vivo* findings of tricellulin dislocalization in *Occ^−/−^* cochlea support the former one. The mutations in *TRIC*, a gene encoding human tricellulin, were reported to be responsible for hereditary deafness DFNB49 (Riazuddin et al., 2006), and knockin mice mimicking human mutation exhibit deafness associated with progressive hair cell degeneration (Nayak et al., 2013), as observed in *Occ^−/−^*, claudin-14 and claudin-9 mutant mice. The DFNB49 mutations of tricellulin result in the immature termination and cause the dislocalization of tricellulin (Nayak et al., 2013). Recently, another tricellular component, ILDR1, is also responsible gene of hereditary deafness DFNB42 (Borck et al., 2011). ILDR1 is one of angulin family proteins, which are required for the localization of tricellulin at tricellular contacts (Masuda et al., 2011; Higashi et al., 2013). Taken together, the dislocalization of tricellulin is likely to be the cause of deafness in *Occ^−/−^* mice.

## MATERIALS AND METHODS

### Antibodies

We previously raised and characterized rat anti-mouse occludin mAb (MOC37), rabbit anti-mouse claudin-14 pAb, rat anti-mouse radixin mAb (R21), rat anti-mouse ZO-1 mAb and rat anti-mouse tricellulin mAb (Saitou et al., 1997; Kitajiri et al., 2004c; Kitajiri et al., 2004b; Hirao et al., 1996; Ikenouchi et al., 2005). Rabbit anti-ZO-1 pAb was purchased from Zymed Lab (San Francisco, California, USA). Rabbit anti-human myosin VIIa pAb was provided by Dr Tama Hasson (University of California, Los Angeles, CA) (Hasson et al., 1995).

### Generation of *Occ^−/−^* mice

*Occ^−/−^* mice were generated as previously reported (Saitou et al., 2000). Two independent mouse J1 ES clones (129/Sv), in which the occludin gene was correctly disrupted, were injected into C57BL/6 blastocysts, and the resulting chimeras were mated with C57BL/6 mice (Doi et al., 1999).

### Auditory Brainstem Response (ABR) measurements

ABR measurements were performed in a soundproof room according to the method described previously (Zheng et al., 1999; Kitajiri et al., 2004b; Kitajiri et al., 2004a). In general, ABR waveforms were recorded for 12.8 ms at a sampling rate of 40,000 Hz using 50–5,000 Hz filter settings; waveforms recorded from 1,024 stimuli at a frequency of 9 Hz were averaged. ABR waveforms were recorded in decreasing 5-dB SPL intervals from the maximum amplitude until no waveforms could be visualized.

### Vestibulo-Ocular Reflex (VOR) measurements

VOR was measured as described previously (Iwashita et al., 2001; Kitajiri et al., 2004a). Head movements were transduced to DC signals using a small angular velocity sensor (Gyrostar, Murata Corporation, Japan), which was fixed on the turntable. Eye movements were detected by LED and a CCD camera, and eye velocities were calculated online by downloading them onto a computer through a video capture board. Both the head and eye velocity curves were fitted with sinusoidal curves using the least squares criterion, and the gain of eye velocity relative to the head velocity was obtained.

### Immunofluorescence microscopy

Temporal bones were removed from *Occ^+/+^* or *Occ^−/−^* mice, and the round and oval windows were opened, together with the small holes in the cochlear apical turn and superior semicircular canal. The perilymphatic space was gently perfused with 10% trichloroacetic acid (TCA) from the round to oval windows (Hayashi et al., 1999; Kitajiri et al., 2004c). Then the samples were immersed in 10% TCA for 1 hour, washed 3 times with phosphate buffered saline (PBS), and decalcified with 5% EDTA in PBS for 3 days. They were microdissected and mounted on slide glasses for whole mount staining. Some specimens were immersed in 30% sucrose in PBS for 1 day, and frozen in liquid nitrogen. Frozen sections ∼10 m thick were cut from these samples and air-dried on slide glasses. The whole mount samples and frozen sections were treated with 0.2% Triton X-100 in PBS for 15 min, and soaked in 1% bovine serum albumin (BSA) in PBS. The whole mounts and sections were then incubated with primary Abs for 30 min at room temperature. They were then washed three times with PBS, followed by a 30-min incubation with Cy3- or Alexa Fluor® 488-conjugated secondary antibody. After a wash with PBS, they were embedded in 95% glycerol-PBS containing 0.1% paraphenylendiamine and 1% *n*-propylgalate. Fluorescence images were obtained with a confocal microscope (model LSM 510 META; Carl Zeiss MicroImaging, Inc.) or with a DeltaVision optical sectioning microscope (version 2.10; Applied Precision, Inc.), equipped with an Axioplan2 (Plan Apochromat 63/1.40 NA oil immersion objective; Carl Zeiss MicroImaging, Inc.) or IX70 (PlanApo 60/1.40 NA oil immersion objective; Olympus) microscope, respectively.

### TUNEL method

Apoptotic cells were detected by the terminal deoxynucleotidyl transferase-mediated dUTP nick-end labeling (TUNEL) method. After microdissection for whole mount staining, the specimens were permeabilized in 0.5% Triton X-100 in PBS for 30 min. TUNEL staining was performed using an Apoptag Fluorescein Direct In Situ Apoptosis Detection Kit (Intergen Company, Purchase, NY) according to the supplier's instruction. The specimens were observed using a DeltaVision system.

### Scanning electron microscopy

Temporal bones obtained from *Occ^+/+^* or *Occ^−/−^* mice were fixed using perilymphatic perfusion as described above with 1% glutaraldehyde in 0.1 M phosphate buffer (pH 7.2). They were then washed with phosphate buffer and post-fixed in 1% OsO_4_ for 2 hours, after which they were once again treated with perilymphatic perfusion. The organ of Corti or crista ampullaris was microdissected, dehydrated, critical-point dried, sputter coated, and observed by scanning EM (model S-800 microscope; Hitachi Co.).

### Ultrathin-section electron microscopy

Samples were fixed as described above, dehydrated with ethanol and embedded in Polybed 812 (Polyscience). Ultrathin sections were cut, doubly stained with uranyl acetate and lead citrate and viewed with a JEM 1010 transmission electron microscope (JEOL).

### Explant culture

Cochleae from the temporal bones of 3-day-old *Occ^+/+^* or *Occ^−/−^* mice under deep anesthesia with ether were dissected in PBS without calcium. After microdissection, cochleae were placed onto the sterile filter membrane (Millicell, 12 mm, Millipore, MA, USA) in standard medium composed of Minimum Essential Medium (Invitrogen Corp., CA, USA) added with 3 g/l glucose and 0.3 g/l penicillin G potassium salt (Nacalai Tesque Inc., Kyoto, Japan) into a 24 well culture plate (Asahi Techno Glass Corp., Tokyo, Japan). Cochleae were then incubated at 37°C in a humidified atmosphere of 95% air and 5% CO_2_.

### Tracer permeability assay

Temporal bones were removed from 6-week-old mice, and the round and oval windows were opened in PBS containing 1 mM CaCl_2_. As mentioned above, the perilymph space was carefully perfused with 100 µl of 10 mg/ml EZ-Link^TM^ Sulfo-NHS-LC-Biotin (Pierce Chemical Co., Rockford, IL) in PBS containing 1 mM CaCl_2_ for 5 minutes (Chen et al., 1997), followed by perfusion for 5 times with PBS containing 1 mM CaCl_2_. The temporal bones were then fixed by perilymphatic perfusion with 10% TCA for 1 hour, and processed for immunofluorescence microscopy. The distribution of injected biotin tracer was visualized by incubating frozen sections with streptavidin-FITC (Oncogene Res. Products, Boston, MA) for 30 minutes.

Experimental protocols and animal care were approved by the Institute of Laboratory Animals Animal Research Committee, Graduate School of Medicine, Kyoto University.

## Supplementary Material

Supplementary Material
