# Supplementary material for: Deafness in occludin-deficient mice with dislocation of tricellulin and progressive apoptosis of the hair cells
Source: Biol Open. 2014 Jul 25;3(8):759–66. doi: 10.1242/bio.20147799 (PMC4133728; doi:10.1242/bio.20147799)
Supplement: Supplementary Material [file supp_3_8_759__index.html]

Deafness in occludin-deficient mice with dislocation of tricellulin and progressive apoptosis of the hair cells — Deafness in occludin-deficient mice with dislocation of tricellulin and progressive apoptosis of the hair cells — Supplementary Material 

# Deafness in occludin-deficient mice with dislocation of tricellulin and progressive apoptosis of the hair cells

## bio.20147799 Supplementary Material

**Files in this Data Supplement:**

- Supplementary Material - Shin-ichiro Kitajiri et al. doi: 10.1242/bio.20147799
