# Supplementary material for: Deafness in occludin-deficient mice with dislocation of tricellulin and progressive apoptosis of the hair cells
Source: Biol Open. 2014 Jul 25;3(8):759–66. doi: 10.1242/bio.20147799 (PMC4133728; doi:10.1242/bio.20147799)
Supplement: Supplementary Material [file supp_bio.20147799_bio.20147799-s1.pdf]

Biology Open (2014) 000, 1–8 doi:10.1242/bio.20147799

**Supplementary Material**
**Shin-ichiro Kitajiri et al. doi: 10.1242/bio.20147799**

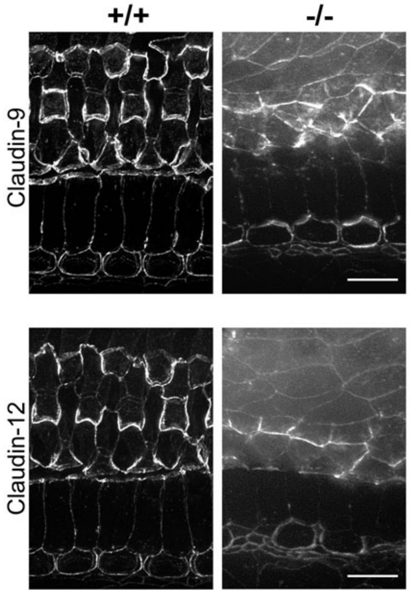


**Fig. S1. Expression of claudin-9 and -12 in the organ of Corti.** The whole mount immunofluorescence micrographs for claudin-9 and claudin-12 of the *Occ*$^{+/+}$ and *Occ*$^{-/-}$ organ of Corti in P15 mice. Claudin-9 and claudin-12 were expressed normally at the junctional complex along the apical borders of the organ of Corti even in *Occ*$^{-/-}$ mice. Scale bars: 10 µm.

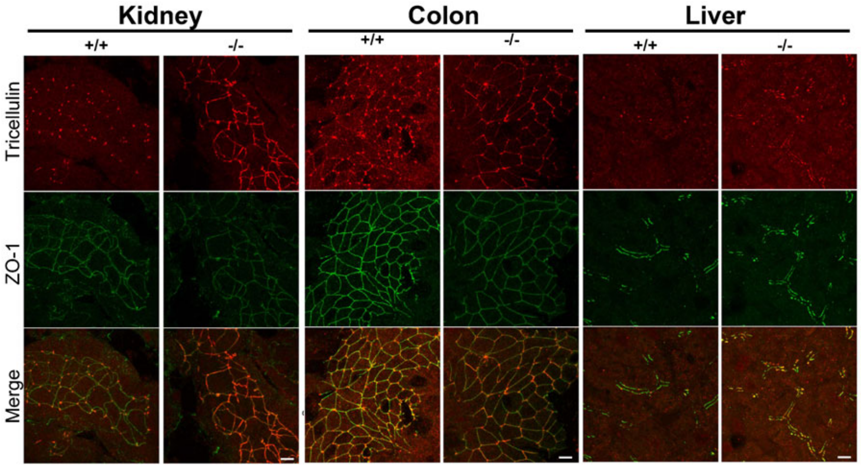


**Fig. S2. The dislocalization of tricellulin in other tissues (kidney, colon and liver) of *Occ*$^{-/-}$ mice.** Kidney, colon and liver of 6-week-old *Occ*$^{+/+}$ and *Occ*$^{-/-}$ mice were labeled with anti tricellulin (red) and anti zo-1 antibodies. In *Occ*$^{+/+}$ tissues tricellulin is predominantly concentrated in tTJs, whereas in all occludin-deficient tissues tricellulin is found in bTJs in addition to tTJs. Scale bars: 5 µm.
